# Profiles of control, value and achievement emotions in primary school mathematics lessons

**DOI:** 10.1111/bjep.12768

**Published:** 2025-03-25

**Authors:** Wendy Symes, Stephanie Lichtenfeld, Peter Wood, David W. Putwain

**Affiliations:** ^1^ Institute for Psychology in Education University of Münster Münster Germany; ^2^ School of Education Liverpool John Moores University Liverpool UK

**Keywords:** achievement emotions, control‐value theory, mathematics, primary school

## Abstract

**Background:**

Achievement emotions are important for mathematical achievement. However, it is currently unclear how specific combinations of emotions—and their associated control and value appraisals—relate to mathematics performance, especially in younger students.

**Aims:**

The aims of this study were to (i) identify heterogeneous profiles of control, value and achievement emotions (enjoyment, boredom and anxiety) experienced during primary‐school mathematics lessons, and to explore how profile membership related to (ii) mathematics test scores and (iii) gender.

**Sample:**

Our sample comprised 883 students (50% girls, *M*_age_ = 9.34 years, *SD* = .48) from 23 primary schools in England.

**Methods:**

Data were collected longitudinally over one academic year. Students completed mathematics tests at T_1_ and T_3_, and self‐reported their control, value and achievement emotions in mathematics lessons at T_2_. A latent profile analysis was conducted to identify profiles of appraisals and emotions. To validate the profiles, T_3_ mathematics test scores and gender were included as covariates of profile membership.

**Results:**

Three profiles were identified: The *Positive* profile, *Negative* profile and *Mixed* profile. Students in the *Positive* profile had significantly higher mathematics test scores at T_3_ than students in the *Mixed* profile. Being a girl increased the likelihood of belonging to the *Mixed* or *Negative* profile relative to the *Positive* profile.

**Conclusions:**

Primary school students' control and value appraisals and achievement emotions co‐occur in line with the theoretical assumptions of CVT. Combinations of emotions should be considered when exploring the impact of emotions on student learning and achievement in mathematics.

## INTRODUCTION

Functional mathematics skills, such as basic arithmetic, are important for the economic well‐being of individuals (Hanushek & Woessmann, 2008) and countries (Kärkkäinen & Vincent‐Lancrin, 2013). Thus, it is concerning that around 27% of primary school students in England leave primary school without reaching the expected minimum standard in mathematics (Department for Education, 2023), with a similar percentage of students (26%) leaving secondary school without achieving a pass grade (a score of 4 or above) in the subject (Department for Education, 2022). These findings are not unique to England, with 19% of students in the United States finishing elementary school without basic mathematics skills (National Center for Educational Statistics, 2019). Understanding the factors that influence the development of mathematics competence is therefore crucial to increasing the number of students attaining mathematical literacy. One factor that has been shown to relate to the development of mathematics skills is affect (Passolunghi et al., 2019), and students' self‐reported emotions towards mathematics may explain as much variance in their mathematics grades as cognitive ability (Pekrun, Marsh, Suessenbach, et al., 2023).

According to control‐value theory (CVT; Pekrun, 2006; Pekrun, Marsh, Elliot, et al., 2023) achievement emotions—the emotions experienced in learning and testing situations—arise from students' appraisals of control and value. More specifically, distinct combinations of control and value appraisals are theorized to give rise to specific achievement emotions (Pekrun, 2006). Researchers have started to use person‐centred approaches to identify groups of students who differ in the type and strength of achievement emotions experienced (Jarrell et al., 2016; Lee & Chei, 2020; Parker et al., 2021; Tze et al., 2022). However, relatively few studies have explored emotions experienced in mathematics lessons, especially in primary school. Furthermore, the studies that have (Karamarkovich & Rutherford, 2021; Mata et al., 2022) have not considered how profiles of different mean levels of emotions (e.g. high in one emotion and low in another) relate to control and value appraisals. We addressed these gaps in our study by using latent profile analysis to identify profiles of control and value appraisals, and enjoyment, boredom and anxiety in mathematics lessons in a large sample of primary school students. In doing so, we provide a robust test of control‐value theory in the mathematics context.

### Achievement emotions and the role of appraisals

Primary school students can feel a range of emotions about their lessons and schoolwork (Raccanello et al., 2018), including in mathematics (Bieleke et al., 2023) such as enjoyment, boredom and anxiety (Lichtenfeld et al., 2012). In control‐value theory (CVT; Pekrun, 2006), the type and strength of an emotion experienced by a student in any given achievement setting are linked to their control and value appraisals of that situation. Control appraisals are defined as students' subjective judgements regarding the likelihood of completing an activity successfully and can include self‐efficacy and self‐concept (Pekrun, 2006). Value appraisals are the subjective judgements of the intrinsic or extrinsic value of an activity (Pekrun et al., 2011), with intrinsic value being particularly salient (Pekrun, Marsh, Elliot, et al., 2023). Specific achievement emotions are posited to arise from specific combinations of control and value appraisals (Pekrun, 2006; Pekrun, Marsh, Elliot, et al., 2023). For example, enjoyment should arise from high control and high value, boredom from either high or low control and low perceived value, and anxiety from high value but low control (Pekrun, 2006). These propositions have been supported in variable‐centred studies examining the emotional experiences of primary school students in mathematics lessons (Putwain et al., 2021; Putwain, Pekrun, et al., 2018).

Previous research examining the linkages between control and value appraisals and achievement emotions (such as those cited above) has typically used longitudinal designs and included control and value appraisals as predictors of achievement emotions. However, although CVT positions control and value appraisals as temporal antecedents of achievement emotions, this may not always be the case (Pekrun, 2006). For example, in contexts familiar to a student, such as typical classroom instruction, achievement emotions may become habitualized and conscious appraisals may not be needed to elicit them (Pekrun, 2006). Furthermore, given that the relations between appraisals and emotions are assumed to be reciprocal (Forsblom et al., 2022), control and value appraisals and emotions are likely to become closely aligned over time. Thus, it could be theorized that—on the trait level at least—students' appraisals and emotions would co‐occur. In addition, although specific control and value appraisals are associated with the experience of individual achievement emotions, it is likely that the same set of control and value appraisals will co‐occur with distinct combinations of emotions. For example, a student with high control and high value is likely to experience high enjoyment alongside low boredom and anxiety. We list these theoretically plausible combinations of control, value and achievement emotions in Table 1. We focus on enjoyment, boredom and anxiety since these are the most commonly explored achievement emotions in the primary school mathematics context (Lichtenfeld et al., 2012).

**TABLE 1:** Theoretically plausible profiles of control and value appraisals and achievement emotions.

Profile	Appraisals		Emotions		
	Control	Value	Enjoyment	Boredom	Anxiety
Positive	High	High	High	Low	Low
Mixed	High	Low	Low/Moderate	Low/Moderate	Low
Negative (Anxiety)	Low	High	Low	Low	High
Negative (Boredom)	Low	Low	Low	High	Low

### Prior research examining profiles of achievement emotions in primary school mathematics lessons

Recently, researchers have begun using person‐centred approaches to analysis to explore interindividual differences in combinations of achievement emotions (see, for example, Tze et al., 2022). Of these studies, two (Karamarkovich & Rutherford, 2021; Mata et al., 2022) have focused on primary‐school mathematics students. Karamarkovich and Rutherford (2021) identified four emotion profiles in a large sample of primary school students: two positive profiles, one negative profile and one mixed profile. Furthermore, they found that students belonging to profiles characterized by more negative emotions reported lower control and value than students in more positive profiles. However, the researchers did not measure enjoyment or anxiety, and students were only asked to identify the emotions they experienced most in mathematics lessons. Therefore, the profiles were not characterized by high levels of some emotions and low levels of others and cannot be compared to the profiles in Table 1. Furthermore, control and value were not included in the profiles but rather were used as predictors of profile membership. Thus, the study only offers limited support for the theorized profiles of control, value and achievement emotions presented in Table 1.

Mata et al. (2022) identified three heterogeneous profiles of enjoyment, boredom and anxiety in a smaller sample of primary school students in mathematics lessons. In line with the profiles presented in Table 1, they identified a positive profile characterized by high enjoyment and low anxiety and boredom, as well as a negative profile characterized by low enjoyment, moderate anxiety and high boredom. This profile was similar to the ‘negative (boredom)’ profile in Table 1, but with moderate, rather than low, anxiety. Finally, they identified a moderate profile characterized by moderate enjoyment and low boredom, but, unlike the ‘mixed’ profile in Table 1, moderate levels of anxiety as well. Since the researchers did not include measures of control or value in their study, it was not possible to test whether the profiles of emotions related to control and value appraisals in the expected ways. Thus, the study provides only partial support for the theorized profiles in Table 1.

The only study to date that has explored profiles of control, value, enjoyment, boredom and anxiety was conducted with university students taking an online course (Parker et al., 2021). The researchers identified three profiles that broadly supported the theorized profiles in Table 1. More specifically, the largest profile was characterized by high control and value appraisals coupled with high enjoyment and low boredom, similar to the ‘positive’ profile. The second profile was characterized by low control and high boredom, similar to the ‘negative (boredom)’ profile, but it was additionally characterized by high anxiety as well, similar to the ‘negative (anxiety)’ profile. The third profile was characterized by low value and high boredom, similar to the ‘negative (boredom)’ profile. Whilst this study provides some support for the existence of the theorized profiles in Table 1, it remains to be tested whether the same profiles would be identified in a sample of primary school mathematics students.

### Achievement emotions and mathematics achievement

Understanding more about the emotional experiences of primary school students in their mathematics lessons is important because, according to CVT, students' achievement emotions are linked to their learning and achievement (Pekrun, 2006). This assumption has been supported by variable‐centred studies that have shown that higher enjoyment in mathematics is positively related to mathematics achievement, including grades and test scores, whilst higher boredom or anxiety are negatively related (Lichtenfeld et al., 2012, 2023; Putwain et al., 2021; Putwain, Becker, et al., 2018). CVT (Pekrun, 2006) proposes that achievement emotions relate to academic performance via their influence on students' self‐regulatory, cognitive and motivational strategies. In support of this, previous research has shown that positive achievement emotions (e.g. enjoyment) relate positively to students' use of self‐regulated learning strategies, whilst negative emotions (e.g. boredom and anxiety) relate negatively to the same outcomes (Ahmed et al., 2013). Accordingly, students' achievement emotions are theorized to influence students' academic achievement over and above prior achievement (Pekrun, 2006). This assumption has been supported in research examining the reciprocal relations between primary school students' mathematics achievement and achievement emotions (Putwain et al., 2022). However, until recently, it has been less clear how combinations of achievement emotions relate to performance.

With regard to the primary school mathematics context, Mata et al. (2022) examined how membership in the three identified profiles of enjoyment, boredom and anxiety related to mathematics competence (including test scores and reported grades). They found that the students in the positive profile outperformed students in the negative profile and the moderate profile, supporting the assumptions of CVT that the experience of more positive achievement emotions relates positively to performance. However, they additionally found that students in the moderate profile outperformed students in the negative profile, despite having higher anxiety. Thus, the combination of emotions (e.g. moderate anxiety in combination with higher enjoyment and lower boredom) may buffer students ‐to some extent‐ from the negative effects of anxiety on performance. Similarly, Karamarkovich and Rutherford (2021) found that students belonging to the more positive emotion profiles had higher maths achievement than students in the more negative or mixed profiles. In addition, Karamarkovich and Rutherford (2021) found that profile membership mediated the relations between control and value appraisals and achievement. However, since they did not include control and value within the profiles themselves, and since Mata et al. (2022) did not measure control and value appraisals, it remains untested how membership in profiles of control, value, and achievement emotions relates to mathematics achievement in primary school students. However, in their study involving university students, Parker et al. (2021) found that students belonging to the high control, high enjoyment profile outperformed students in the other two, more negative profiles. Thus, it could be assumed that primary school students belonging to a profile such as the ‘positive’ profile in Table 1 would perform better in mathematics tests than students in the other three profiles. Further research is needed to test this assumption.

### Gender and profile membership

CVT (Pekrun, 2006) does not explicitly consider how gender might influence students' achievement emotions. However, it is plausible that boys and girls may have different affective experiences in mathematics lessons due to gender differences in their appraisals of control and value (Frenzel et al., 2007). It is well‐established that, in Western contexts at least, boys typically report higher levels of control and/or value for mathematics than girls, including in primary school (Eccles et al., 1993; Lichtenfeld et al., 2012; Putwain et al., 2021). Furthermore, studies have shown that boys may also be more likely than girls to experience positive achievement emotions in mathematics, such as enjoyment, and less likely to experience negative emotions such as anxiety and boredom (Frenzel et al., 2007; Lichtenfeld et al., 2012; Putwain et al., 2021). Consequently, it could be assumed that gender would relate to membership in the profiles outlined in Table 1, with girls having a higher likelihood of belonging to more ‘negative’ emotion and appraisal profiles. However, research findings with primary school students have been contradictory. Whilst Karamarkovich and Rutherford (2021) reported that boys had a lower likelihood than girls of belonging to more negative profiles of mathematics‐related emotions, Mata et al. (2022) found no gender differences in profile membership. Further research is needed regarding the relations between gender and profile membership.

### The current study

The aim of our research was to identify profiles of control, value and three achievement emotions (enjoyment, boredom and anxiety) in mathematics lessons in a sample of primary school students, and to examine covariates of these profiles. More specifically, we included gender as a predictor of profile membership and mathematics achievement as an outcome. This approach allowed us to validate our final model and provide support for CVT by testing if the covariates related to the identified profiles in theoretically expected ways (Asparouhov & Muthén, 2014). In addition, to test the theoretical assumption of CVT that students' achievement emotions should influence students' mathematics achievement over and above prior mathematics achievement (Pekrun, 2006), we controlled for prior mathematics achievement. Thus, our study provides a robust test of CVT in the primary‐school mathematics context.

Our study answered the following research questions and tested the following hypotheses:

Research question 1: Which profiles of mathematics‐related control, value, enjoyment, boredom and anxiety do students belong to in their penultimate year of primary school? *Hypothesis 1*: Drawing on CVT and related empirical findings, we predicted that we would identify profiles such as those suggested in Table 1, namely positive, negative or mixed profiles.

Research question 2: How does membership in these profiles relate to mathematics achievement, controlling for prior mathematics achievement? *Hypothesis 2*: Drawing on CVT and related empirical findings, we assumed that mathematics achievement would be highest in students belonging to more ‘positive’ profiles, and lowest in students belonging to more ‘negative’ profiles, even after controlling for prior mathematics achievement.

Research question 3: How does gender relate to profile membership?

Based on the mixed previous findings, we did not make specific hypotheses about gender differences.

## METHOD

### Sample

The participants in this study were *N* = 883 year 5 students from 23 primary schools in England (443 girls and 440 boys; *M*age 9.34 years, *SD* = .48) in their penultimate year of primary school. Most of the sample was White (71%), followed by Asian (19%), Black (4%), other or multi‐ethnic heritage (5%) and Chinese (1%). The sample was drawn from a larger dataset of *N* = 1242 students (609 girls and 633 boys; *M*age 9.34 years, *SD* = 0 .49) participating in a 4‐wave longitudinal study exploring primary school students' experiences in mathematics lessons over one academic year. An overview of the project, including all constructs investigated, measures used and final dataset, can be accessed online via the following link: https://osf.io/5tu4m/. In our study, we focused on data collected in waves 2, 3 and 4 (hereafter referred to as T_1_, T_2_ and T_3_ respectively). Participants were included if control, value or achievement emotion data was available for them at T_2_.

#### Control and value appraisals

Control was measured at T_2_ using four self‐concept items from the *Academic Self‐Description Questionnaire II* (Marsh, 1992). All items were worded to refer to mathematics lessons. A sample item is ‘I can learn things quickly in maths lessons’. Participants responded on a 5‐point scale from 1 = ‘strongly disagree’ to 5 = ‘strongly agree*’*. Internal consistency was good (⍺ = .82).

Three types of value—intrinsic value, attainment value and utility value—were measured using 4 items each from the *Michigan Study of Adolescent Life Transitions* scales, adapted for use with primary school students (Putwain, Pekrun, et al., 2018). All items were worded to refer to mathematics lessons. Sample items include ‘I am interested in learning maths’ (intrinsic value), ‘I want to get good marks in maths’ (attainment value) and ‘Maths will help me later in life’ (utility value). Participants responded on a 5‐point scale from 1 = ‘strongly disagree*’* to 5 = ‘strongly agree*’*. Internal consistency for each subscale was acceptable or good (intrinsic value: ⍺ = .89; attainment value: ⍺ = .80; utility value: ⍺ = .76).

#### Achievement emotions

Enjoyment, boredom and anxiety in mathematics were measured at T_2_ using 4 items each from the *Achievement Emotions Questionnaire*—*Elementary School* (AEQ‐ES, Lichtenfeld et al., 2012). All items were worded to refer to mathematics lessons and adapted to the school context in England (e.g. ‘class’ was changed to ‘lesson’). Sample items include ‘I look forward to maths lessons’ (enjoyment), ‘I find maths lessons so boring I would rather do something else’ (boredom) and ‘When I think about maths lessons, I get nervous’ (anxiety). Participants responded on a 5‐point scale from 1 = ‘not at all*’* to 5 = ‘very much’. Internal consistency for each subscale was good (enjoyment: ⍺ = .94; boredom: ⍺ = .92; anxiety: ⍺ = .84).

#### Mathematics achievement

Mathematics achievement was assessed at T_1_ and T_3_ using tests developed specifically for the larger project from which the data for this study was drawn. Test items were selected from six National Curriculum Test mathematics reasoning papers (2016–2018) taken by students in England at the end of primary school (year 6). These tests are broadly regarded as valid and reliable (e.g. see Bew, 2011; Ofqual., 2010), and they cover the mathematics curriculum taught in England between school years 3 and 6. Two primary school teachers unrelated to the research project were asked to select items from the past papers that reflected the participants' school stage (i.e. year 5) and the different elements of the curriculum they were following (e.g. ratio and proportion, simple algebra, geometry, fractions and statistics and measurement). The selected items were then randomly assigned to either of the two mathematics tests. The T_1_ test had 19 questions, whilst the T_3_ test had 16 questions. Students could gain 1 or 2 points per question, with a maximum score of 20 per test. Students were allowed 40 minutes to complete the test and could use paper and pencil to assist their working out. Students were not informed of their test scores. Internal consistency at both time points was acceptable or good (T_1_ ⍺ = .78; T_3_ ⍺ = .85). For further information regarding the mathematics tests used in this study see Putwain et al. (2022).

### Procedure

Once ethical approval for the study had been received from the third and fourth authors' institutional research ethics committee (19/EHC/01), schools were recruited to participate in the study. For more details on the recruitment process, see Putwain et al. (2022). Data was collected in the participants' classrooms during the typical school day by their teachers reading from a standardized script. Along with instructions, this standardized script emphasized to students that participation in the study was voluntary, that they did not have to participate, and that they were free to withdraw from the study without giving a reason. Students completed all questionnaire and test items online, and all responses were anonymous. The T_1_ mathematics test was completed at the end of the first term of the academic year (December 2018). T_2_ control, value and achievement emotions data were collected around 6 months later towards the end of the academic year (June 2019). The T_3_ mathematics test was completed approximately 1 week after T_2_ data collection was complete.

The number of participant responses for each variable is shown in Table 2. A Little's test was statistically significant (*p* < .01), indicating that the assumption of data missing completely at random (MCAR) was not met. Participants with missing maths test data at T_1_ reported significantly lower boredom at T_2_ *t*(875) = −2.029, *p* < .05. Participants with missing mathematics test data at T_3_ had significantly lower T_1_ mathematics test performance *t*(752) = −3.681, *p* < .05 and significantly lower intrinsic value *t*(881) = −4.351, *p* < .01, attainment value *t*(881) = −2.964, *p* < .01 and enjoyment *t*(875) = −3.495, *p* < .05 at T_2_, as well as significantly higher boredom *t*(875) = 3.185, *p* < .05. All other differences were non‐significant. When the cause of missingness can be identified, data can be treated as missing at random (MAR) and handled using full‐information maximum likelihood estimation (Nicholson et al., 2017). Accordingly, missing data for the latent profiles was handled in Mplus using FIML.

**TABLE 2:** Descriptive statistics and correlations for all study variables.

Variable	*n*	*M*	*SD*	1	2	3	4	5	6	7	8	9	10
1. T_2_ Control	883	3.64	.89	_									
2. T_2_ Intrinsic value	883	3.95	1.05	.**64**	**_**								
3. T_2_ Attainment value	883	4.40	.76	.**50**	.**54**	**_**							
4. T_2_ Utility value	883	4.36	.72	.**48**	.**54**	.**64**	**_**						
5. T_2_ Enjoyment	877	3.85	1.19	.**64**	.**88**	.**50**	.**47**	**_**					
6. T_2_ Boredom	877	2.11	1.22	**−.42**	**−.75**	**−.38**	**−.39**	**−.75**	**_**				
7. T_2_ Anxiety	877	2.02	1.07	**−.42**	**−.45**	**−.25**	**−.31**	**−.43**	.**57**	**_**			
8. T_1_ Maths Test	754	4.91	3.52	.**36**	.**25**	.**17**	.**20**	.**23**	**−.23**	**−.32**	**_**		
9. T_3_ Maths Test	654	6.94	3.88	.**36**	.**22**	.**17**	.**19**	.**22**	**−.24**	**−.38**	.**70**	_	
10. Gender				**−.14**	**−.14**	.02	.02	**−.14**	.**10**	.**09**	**−.10**	**−.11**	_

*Note*: Significant correlations are in **bold**. All significant correlations were significant to *p* < .01. For Gender, boys = 0, girls = 1.

### Statistical analysis

All statistical analyses were conducted using Mplus version 8.9. We used T_2_ control, intrinsic value, attainment value, utility value, enjoyment, boredom and anxiety mean scores for the latent profile analysis. We ran a series of latent profile models using the Mplus default settings whereby profile means, but not variances, were allowed to vary. We accounted for the nestedness of the data (students nested in school) using the ‘type = complex’ command. To decide the best‐fitting model we considered model fit statistics, namely the Bayesian Information Criterion (BIC), the Sample‐size adjusted Bayesian Information Criterion (aBIC) and the Vuong‐Lo‐Mendall‐Rubin likelihood‐ratio test (VLMR‐LRT), alongside smallest profile size and theoretical plausibility and interpretability (Ferguson et al., 2020; Nylund‐Gibson et al., 2023; Wickrama et al., 2022). With regards to the smallest profile size, we did not consider models where the smallest profile size comprised less than 5% of the sample and stopped running new models once this threshold had been reached (Ferguson et al., 2020). With regards to theoretical plausibility and interpretability, the theoretical relevance, number and uniqueness of the identified profiles were examined. Models with fewer and more distinct profiles were favoured (Wickrama et al., 2022). Entropy value was used to assess the classification accuracy of the chosen model (Nylund‐Gibson et al., 2023). Finally, we used the manual three‐step procedure outlined by Asparouhov and Muthén (2014) to include T_3_ mathematics achievement as an outcome (controlling for T_1_ mathematics achievement), and the R3STEP command to include gender as a predictor of profile membership.

## RESULTS

### Descriptive statistics

Descriptive statistics and correlations of mathematics control, values, achievement emotion, maths test scores and gender are displayed in Table 2. Control, values, enjoyment and test scores were all positively correlated. Boredom and anxiety were positively correlated with each other but negatively correlated with control, values, enjoyment and test scores. Girls had lower control, intrinsic value, enjoyment and test scores, and higher boredom and anxiety than boys.

### Latent profile analysis

Fit statistics for the latent profile models with one to five profiles are displayed in Table 3. Despite having the lowest BIC and aBIC, we did not select the five‐profile model as the final model. This was because the smallest profile comprised less than 5% of the sample, and the VLMR‐LRT test indicated that it was not significantly better than the four‐profile model. The VLMR‐LRT was significant for the two, three, and four profile models. Although the four‐profile model had the lowest BIC and aBIC scores of the three models, we selected the three‐profile model as the final model. Whilst the profiles identified in the two‐, three‐, and four‐profile models were theoretically plausible, the four‐profile model included two classes that were not qualitatively distinct from each other or the other two profiles, whilst the two‐profile model did not include a theoretically expected ‘negative’ profile. Accordingly, we chose the three‐profile model as the final model. The entropy score of .92 indicated that the chosen model had high classification accuracy (Clark & Muthén, 2009).

**TABLE 3:** Latent profile analysis model fit statistics.

Model	LL	BIC	aBIC	VLMR‐LRT *p* value	Smallest profile (%)
1	−8539.44	17,173.85	17,129.39		
2	−7342.46	14,834.16	14,764.29	<.001	30
3	−6862.82	13,929.14	13,833.86	<.05	12
4	−6661.20	13,580.16	13,459.48	<.05	8
5	−6489.54	13,291.12	13,145.03	.173	2

*Note*: *n* = 883. Abbreviations: aBIC, adjusted BIC; BIC, Bayesian Information Criterion; LL, log‐likelihood; VLMR‐LRT, Vuong‐Lo–Mendell–Rubin Adjusted Likelihood Ratio Test.

**FIGURE 1:**
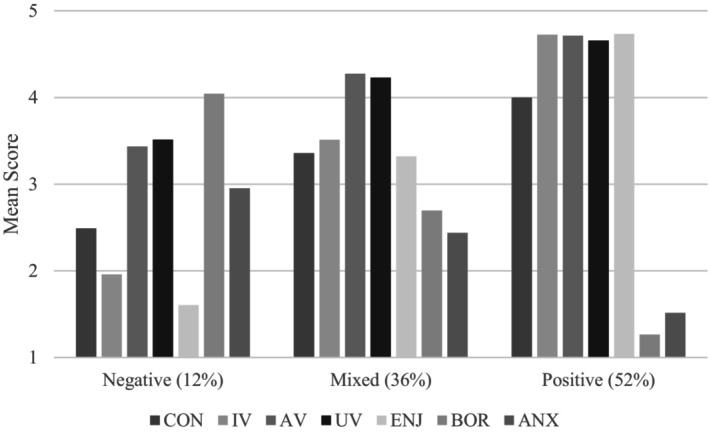
Profile means of students' control, values and achievement emotions in mathematics lessons. CON, control; IV, intrinisc value; AV, attainment value; UV, utility value; ENJ, enjoyment; BOR, boredom; ANX, anxiety.

Profile means for control, values, and achievement emotions are displayed in Figure 1. For comparison, profile means for the two‐ and four‐profile models are provided in Figures S1 and S2 respectively in the Supplemental Material. Profiles differed primarily in levels of intrinsic value, enjoyment, and boredom. Students in the *Positive* profile (*n* = 459) had the highest levels of control, values, and enjoyment, and the lowest levels of boredom and anxiety. Conversely, students in the *Negative* profile (*n* = 108) had the lowest levels of control, values (particularly intrinsic value), and enjoyment, and the highest levels of boredom and anxiety. Finally, students in the *Mixed* profile (*n* = 316) had control, value, and achievement emotions scores that fell roughly in the middle of the other two profiles. In this profile, attainment and utility value were higher than intrinsic value (comparable to the *Negative* profile), and enjoyment was the most strongly reported emotion (comparable to the *Positive* profile).

### Profile membership and mathematics achievement

Once the final model had been selected, we explored how profile membership related to mathematics achievement at T_1_ and T_3_. Profile means for the mathematics tests are presented in Table 4. We controlled for gender in all models.

**TABLE 4:** Profile means of students' mathematics test scores at T_1_ and T_3_.

	Negative	Mixed	Positive
T_1_ Maths Test	3.77	3.89	5.88
T_3_ Maths Test	5.96	5.44	8.09

A Wald test indicated that there was a main effect of profile membership on mathematics test scores at T_1_: χ^2^(2) = 27.95, *p* < .001. Pairwise difference tests indicated that students in the *Positive* profile had significantly higher test scores at T_1_ than students in the *Negative* profile (*z* = 4.20, *p* < .001) and *Mixed* profile (*z* = 4.61, *p* < .001). There was no significant difference between the *Negative* and *Mixed* profiles. A Wald test indicated that there was a main effect of profile membership on mathematics test scores at T_3_: χ^2^(2) = 72.19, *p* < .001. Pairwise difference tests indicated that students in the *Positive* profile had significantly higher test scores at T_3_ than students in the Negative profile (*z* = 3.53, *p* < .001) and *Mixed* profile (*z* = 8.18, *p* < .001). There was no significant difference between the *Negative* and *Mixed* profiles. Finally, A Wald test indicated that the main effect of profile membership on mathematics test scores at T_3_ remained when controlling for T_1_ mathematics test scores: χ^2^(2) = 25.60, *p* < .001. However, only the difference between the *Positive* profile and *Mixed* profile remained significant (*z* = 4.34, *p* < .001). All other differences were non‐significant.

### Gender as a covariate of profile membership

Finally, we explored how gender was related to profile membership. We used the *Positive* profile as the reference profile since it was the largest profile. Gender was significantly related to the likelihood of belonging to the *Negative* profile (*β* = .50, *p* < .05) and the *Mixed* profile (*β* = .51, *p* < .01), relative to the *Positive* profile. More specifically, girls were more likely than boys to belong to the *Negative profile* (OR = 1.65, 95% CI [1.05 2.58]) or the *Mixed* profile (OR = 1.66, 95% CI [1.24 2.23]), relative to the *Positive* profile.

## DISCUSSION

The purpose of this study was to explore heterogeneity in primary school students' control, value, and achievement emotions in mathematics lessons, and to provide a test of CVT in the primary‐school mathematics context. Using latent profile analysis, we identified three profiles characterized by interindividual differences in the included constructs. In line with the theorized profiles presented in Table 1, and previous research with primary school students (Karamarkovich & Rutherford, 2021; Mata et al., 2022) we identified a *Positive* profile, a *Negative* profile, and a *Mixed* profile. The negative profile did not, however, align completely with either of the two theorized negative profiles, as it was characterized by high boredom alongside moderate anxiety. Furthermore, students in this profile reported the highest levels of anxiety as well as boredom. Therefore, we chose to label this the *Negative* profile to reflect the relatively high levels of both negative emotions. There was evidence that profile membership was related to mathematics achievement, with the *Mixed* profile performing significantly worse on the mathematics test than the *Positive* profile at T_3_, when controlling for mathematics performance at T_1_. Finally, gender was found to significantly relate to the likelihood of profile membership, with the likelihood of belonging to the *Mixed* and *Negative* profiles relative to the *Positive* profile increasing for girls. We discuss the study's key findings and implications in the remainder of this section.

### Profiles of control, value, and enjoyment boredom, and anxiety in primary school mathematics lessons

The profiles identified in our study support the theoretical assumptions of CVT regarding relations between control and value appraisals and the experience of achievement emotions (Pekrun, 2006; Pekrun, Marsh, Elliot, et al., 2023). More specifically, and supporting our first hypothesis, higher control and value appraisals occurred alongside higher enjoyment, whilst lower control and/or value appraisals occurred alongside higher boredom and anxiety. The linkages between control, value, and individual achievement emotions in primary school mathematics students have been well established in variable‐centred studies (Putwain et al., 2021; Putwain, Pekrun, et al., 2018). However, the present study is one of only two person‐centred studies to have explored how control and value link to the experience of multiple emotions in primary school students in the mathematics context. Like the previous study (Karamarkovich & Rutherford, 2021) we also found that control and value appraisals relate to the specific constellation of achievement emotions in line with the assumptions of CVT. Our findings additionally support the possibility that, at the trait level at least, control and value appraisals and achievement emotions co‐occur in theoretically expected ways, aligning with previous work with older students (Parker et al., 2021).

However, although we found support for the theorized ‘Positive’ and ‘Mixed’ profiles (see Table 1), our negative profile was a combination of the ‘Negative (boredom)’ and ‘Negative (anxiety)’ profiles. This was similar to the negative profile identified by Mata et al. (2022), which was also characterized by high boredom and moderate anxiety. However, in our study, students in the ‘Negative’ profile also reported the highest levels of anxiety, whereas in the Mata et al. (2022) study, anxiety was higher in the moderate profile. Our negative profile was also similar to the low control—high boredom profile found in Parker et al. (2021), which was characterized by both high boredom and high anxiety, although in our study, boredom was higher than anxiety. One possible reason for these differences is the fact that we included both intrinsic and extrinsic values in our profiles, whereas Parker et al. (2021) only included extrinsic values, and Mata et al. (2022) did not include values.

According to CVT, intrinsic value is particularly relevant for the experience of achievement emotions (Pekrun, Marsh, Elliot, et al., 2023), and this seemed to be the case in our study. More specifically, between‐profile differences in intrinsic value were greater than those of attainment or utility value, and levels of intrinsic value were closely aligned with reported enjoyment and boredom. With regard to anxiety, intrinsic value seemed to play a protective role. Although CVT proposes that anxiety should arise when value is high and control is low (Pekrun, 2006), the highest level of anxiety was experienced in the only profile in which intrinsic value was lower than control (the *Negative* profile). It is possible that if extrinsic values are higher than control, high intrinsic value may protect against anxiety in some way, perhaps by making the learning experience more ‘pleasant’. Further research is needed to test this assumption, and theorizing about expected profiles of emotions may also need to consider the impact of intrinsic and extrinsic value separately.

### Predictors and outcomes of profile membership

In support of our second hypothesis, profile membership related to students' mathematics' achievement, controlling for prior achievement. However, whilst students in the *Positive* profile achieved higher mathematics test scores than students in the *Mixed* profile, they did not do significantly better than students in the *Negative* profile when prior mathematics test scores were controlled for. This finding was surprising given that CVT assumes that both boredom and anxiety should have a negative impact on achievement via their impact on motivation and self‐regulated learning strategies (Ahmed et al., 2013; Camacho‐Morles et al., 2021). Moreover, findings from variable‐centred studies suggest that as negative emotions in primary school mathematics lessons increase, achievement decreases (Lichtenfeld et al., 2023). However, person‐centred studies have reported equivocal findings in the relations between emotion profile membership and achievement (Mata et al., 2022; Parker et al., 2021), indicating that the specific constellation of emotions may be as relevant to performance as the mean levels of each emotion. For example, in Mata et al. (2022) students in the ‘Mixed’ profile outperformed students in the ‘Negative’ profile in mathematics, despite having higher levels of anxiety.

In our study, it is possible that the moderate levels of anxiety in the *Negative* profile may have offset some of the negative impact of high boredom on achievement by providing a ‘push’ to increase effort. For example, in attentional control theory (Eysenck & Derakshan, 2011) it is proposed that anxious individuals may display high motivation (and increased effort) to complete tasks that are perceived as demanding. It is plausible that students in the *Negative* profile would find completing mathematics tasks—such as a mathematics test—demanding, since they also displayed the lowest levels of control. Furthermore, research with secondary school students has shown that increased effort can attenuate the negative relation between anxiety and performance (Putwain & Symes, 2018). Although it was not possible to test the role of effort in our study, our findings nonetheless demonstrate the importance of examining the impact of multiple emotions on performance simultaneously and highlight the suitability of using latent profile analysis to do so.

Finally, we found that gender was related to profile membership. The likelihood of belonging to the *Mixed* or *Negative* profile relative to the *Positive* profile was higher for girls than for boys. More specifically, and in line with previous research (Karamarkovich & Rutherford, 2021), girls were overrepresented in profiles characterized by lower control, value, and enjoyment and higher boredom and anxiety. Given that these two profiles were also related to lower mathematics achievement than students in the *Positive* profile, our findings suggest that appraisals of control and value in mathematics lessons, alongside achievement emotions, may in part contribute to the well‐documented gender differences in mathematics achievement that indicate that boys generally outperform girls in mathematics (Olczyk et al., 2023).

### Implications for practice

Taken together, our findings have three important practical implications. First, intrinsic value appeared to be more closely aligned with reported enjoyment and boredom than the two more extrinsic types of value. In addition, it potentially offered protection against anxiety when control was lower than value. Consequently, teachers may wish to focus on raising students' intrinsic value in mathematics lessons. This could be achieved by, for example, providing choices, using appropriately challenging content, and increasing students' sense of belonging in the classroom (Rosenzweig et al., 2022). Second, our findings suggest that improving mathematics achievement may require a holistic approach in which multiple types of emotions (and/or control and value appraisals) are targeted. It cannot be assumed that a decrease in boredom alone, for example, would lead to improved mathematics achievement if enjoyment remains low. Emotional interventions that aim to raise students' awareness of and ability to regulate their achievement emotions may be particularly useful in this regard (Goetz et al., 2005; Raccanello & Hall, 2021). Third, and relatedly, teachers and educational psychologists may wish to assess multiple emotions when identifying students who may benefit most from interventions. For example, if only one emotion (e.g. boredom) is measured, this may mean that students at risk of lower mathematics achievement, such as those in the *Mixed* profile in our study, are not identified.

### Limitations and ideas for future research

When interpreting our findings, it is important to acknowledge the potential limitations of our study. First, although we were interested in identifying which profiles of control, value, and achievement emotions students belonged to, we were unable to determine the stability of these profiles. Future research should consider using a longitudinal design, such as latent transition analysis, to see how profile membership changes over time. Relatedly, students completed the second mathematics tests one week after completing the questionnaires measuring their control, value, and achievement emotions. This limits our ability to draw conclusions about the impact of profile membership on the development of mathematics achievement long term. In future, it may be helpful to measure achievement at multiple time points. In addition, we could not control for cognitive variables such as intelligence, since these were not measured in the larger project from which the data used in our study came. In CVT, such factors are assumed to exert an influence on students' achievement, alongside their achievement emotions. Therefore, it would be important to control for students' cognitive abilities in future research to more clearly demonstrate the impact of control, value, and achievement emotion profile membership on outcomes. Finally, although we included gender as a predictor of profile membership, CVT identifies many aspects of the teaching environment that might shape students' appraisals of control and value, as well as their experience of achievement emotions, such as perceptions of autonomy support or classroom goals. Future research should include covariates such as these to better understand the classroom factors that may influence profile membership, and thus more clearly highlight potential avenues for intervention.

## CONCLUSION

Variable‐centred studies have established that primary school students' affective experiences in mathematics classrooms can influence their mathematics achievement (Lichtenfeld et al., 2023; Putwain, Pekrun, et al., 2018). However, these studies have tended to explore the influence of individual achievement emotions on achievement separately, and it is less clear how multiple emotions may relate to mathematics skills in combination. Our study addressed this by using latent profile analysis to identify heterogeneous profiles of mathematics‐related control, value, enjoyment, boredom, and anxiety in a large sample of primary school students. Our findings indicate that just over half of students belonged to the *Positive* profile, characterized by high control, value and enjoyment, combined with low boredom and anxiety, which is reassuring. However, there was evidence that not all students had such a positive experience. Students in the *Negative* profile reported the opposite pattern, whilst students in the *Mixed* profile had higher control, value, and enjoyment (and lower boredom and anxiety) than students in the *Negative* profile, but lower control, value, and enjoyment (and higher boredom and anxiety) than students in the *Positive* profile. Importantly, profile membership was linked to mathematics achievement, with students in the *Mixed* profile having significantly lower mathematics test scores than students in the *Positive* profile. Our findings suggest that anxiety may buffer the negative effects of high boredom on mathematics achievement and also point to the importance of intrinsic value in the experience of achievement emotions. Teachers may wish to consider ways to increase students' intrinsic value during mathematics lessons, especially in girls, who were less likely to belong to the *Positive* profile than boys.

## AUTHOR CONTRIBUTIONS

**Wendy Symes:** Conceptualization; data curation; formal analysis; methodology; writing – original draft. **Stephanie Lichtenfeld:** Formal analysis; methodology; writing – review and editing. **Peter Wood:** Funding acquisition; investigation; project administration. **David W. Putwain:** Investigation; data curation; funding acquisition; project administration; writing – review and editing.

## CONFLICT OF INTEREST STATEMENT

The authors confirm that there is no conflict of interest for this article.

## Supporting information


Data S1.

## Data Availability

This project was registered with the Center for Open Science. Project materials and the project dataset can be accessed online at: https://osf.io/5tu4m/.
