# Supplementary material for: Profiles of control, value and achievement emotions in primary school mathematics lessons
Source: Br J Educ Psychol. 2025 Mar 25;95(3):888–902. doi: 10.1111/bjep.12768 (PMC12319179; doi:10.1111/bjep.12768)
Supplement: Supplementary file 1 — Data S1. [file BJEP-95-888-s001.docx]

**Supplemental Material**

**Figure S.1**

*Profile Means of Students’ Control, Values and Achievement Emotions in Mathematics Lessons for the Two-Class model*

*Note*. CON = control; IV = intrinsic value; AV = attainment value; UV = utility value; ENJ = enjoyment; BOR = boredom; ANX = anxiety

**Figure S.2**

*Profile Means of Students’ Control, Values and Achievement Emotions in Mathematics Lessons for the Four-Class Model*

*Note*. CON = control; IV = intrinsic value; AV = attainment value; UV = utility value; ENJ = enjoyment; BOR = boredom; ANX = anxiety
